# Survival benefits of immunotherapy combined with chemoradiotherapy in metastatic colorectal cancer: an analysis based on data from the SEER database

**DOI:** 10.1515/med-2026-1416

**Published:** 2026-04-27

**Authors:** Jie Zhang, Weijuan Jiang

**Affiliations:** Department of Radiotherapy, Jingjiang People’s Hospital Affiliated to Yangzhou University, Jingjiang, Jiangsu, China

**Keywords:** metastatic colorectal cancer, SEER, chemoradiotherapy, immunotherapy, survival, prognosis

## Abstract

**Objectives:**

Chemoradiotherapy (CRT) alone shows limited efficacy in improving outcomes for metastatic colorectal cancer (mCRC) patients. This study aimed to comprehensively evaluate the survival benefits of combining CRT with immunotherapy (CRT + IMT) in this population.

**Methods:**

Data from the SEER database were extracted for mCRC patients diagnosed between 2010–2015 and 2018–2020. Survival outcomes were assessed using Kaplan–Meier analysis, log-rank test, Cox regression, and propensity score matching (PSM) to minimize baseline differences between treatment groups, and multivariable Cox models were constructed based on variables selected by LASSO regression.

**Results:**

A total of 2,451 patients were identified, with 1,888 receiving CRT alone and 563 CRT + IMT. After 1:2 PSM, 1,594 remained. Kaplan–Meier curves showed CRT + IMT significantly improved overall survival (OS, p<0.0001) and cancer-specific survival (CSS, p<0.0001). Multivariable Cox confirmed CRT + IMT as an independent predictor (OS HR=0.54, 95 % CI: 0.44–0.65; CSS HR=0.54, 95 % CI: 0.44–0.66). Favorable factors included distal colon/rectal tumors, surgery, and combined therapy, while older age (>60), advanced stage, N2, and metastasis predicted worse survival. The subgroup analyses revealed a more pronounced benefit in specific cohorts, and significant interactions were observed for factors such as histologic grade and nodal stage.

**Conclusions:**

The combination of CRT and immunotherapy confers a significant survival advantage over CRT alone in patients with mCRC, supporting its potential role in optimized treatment strategies.

## Introduction

Colorectal cancer (CRC) represents the third most common cancer and a major contributor to deaths in the U.S., with 153,020 diagnoses and 52,550 deaths in 2023 [1]. Although advanced screening methods, such as colonoscopy and fecal tests, and surgeries have reduced overall CRC rates in the past decades [2], 3], younger adults increasingly face late-stage diagnoses, particularly in the left colon and rectum [1]. Around 20 % of CRC cases are metastatic at diagnosis, and nearly half of localized cases eventually metastasize. CRC spreads via lymphatic, hematogenous, and local abdominopelvic routes [4]. Individuals with metastatic CRC (mCRC) often lose the opportunity for curative surgery, with a low survival rate. mCRC is primarily treated with surgery, systemic therapies (i.e., cytotoxic chemotherapy, biologics such as growth factor antibodies, emerging immunotherapies), locoregional treatments (such as radiotherapy and hepatic artery infusion), and combinations of these treatments [5], 6]. In the past two decades, more effective treatments have extended median survival for mCRC to 2–3 years. However, mCRC remains incurable, with only a 14 % 5-year survival rate [7], 8]. Developing more efficacious treatments remains a priority.

Since the 2000s, several targeted therapies have proven effective in managing mCRC and have been approved by the FDA, encompassing epidermal growth factor receptor (EGFR), vascular endothelial growth factor receptor (VEGF), and DNA mismatch repair [6]. In the U.S., the standard first-line treatment combines chemotherapy with anti-VEGF or anti-EGFR antibodies, depending on tumor characteristics [9], often combined with radiotherapy. However, most patients experience disease progression within a year [10]. Immune checkpoint inhibitors (ICIs) have demonstrated promise in treating mCRC with mismatch repair deficiency (dMMR) [11], 12]. The 2015 KEYNOTE-016 trial demonstrated the benefits of PD-L1 immunotherapy for dMMR/MSI-H CRC [13]. The CheckMate 142 Phase II trial appraised the efficacy of nivolumab in 74 dMMR/MSI-H mCRC patients, in which the objective response rate (ORR) was 31 % in the ICI group, and 69 % of patients achieved disease control within 12 weeks or longer [12]. KEYNOTE-164 confirmed pembrolizumab’s effectiveness in 124 previously treated cases [14]. On the basis of these findings, the FDA approved both drugs (with or without ipilimumab) for advanced, unresectable, or metastatic CRC. Even though dMMR/MSI-H represents a small subset of mCRC, ICIs have revolutionized the precision treatment of mCRC [15]. However, since 95 % of mCRC cases are microsatellite-stable (MSS), also known as cold tumors [16], ICIs remain less effective for most patients compared to inflamed dMMR/MSI-H tumors [17].

The main challenge in treating metastatic CRC is making “cold” tumors more responsive to immunotherapy. Studies indicate that cytotoxic drugs, anti-angiogenics, targeted therapies, and radiotherapy can lead to immunogenic cell death, which enhances the release of plentiful tumor antigens and pro-inflammatory cytokines, increasing immune infiltration [18], 19]. Combining ICIs with chemotherapy has recently improved outcomes in pMMR/MSS patients with proficient mismatch repair (pMMR) or microsatellite-stable (MSS) tumors. ICIs plus systemic chemotherapy could enhance treatment responses in refractory mCRC, particularly, pMMR/MSS CRC [20], 21]. In a phase Ib, single-arm, multicenter study, Herting et al. unraveled the promising efficacy of pembrolizumab plus modified FOLFOX6 in mCRC, including pMMR/MSS CRC [22]. The AtezoTRIBE trial unveiled that first-line FOLFOXIRI plus bevacizumab and atezolizumab was safe and prolonged progression-free survival (PFS) in previously untreated mCRC [23]. In addition to killing tumors, radiotherapy can also boost immunotherapy through the abscopal effect, enhancing anti-tumor activity at distant sites [24]. Pembrolizumab plus radiotherapy achieved a 4.5 % response rate in 22 MSS/pMMR mCRC patients [25]. These findings suggest that combining ICIs with other anticancer therapies may help overcome drug resistance in MSS/pMMR tumors. Although this approach has not been extensively studied, chemoradiotherapy combined with immunotherapy (CRT + IMT) shows potential for improving treatment outcomes. Investigating its survival benefits is essential for ameliorating the prognosis of mCRC.

The SEER database, a comprehensive national cancer database managed by the U.S. National Cancer Institute, covers demographic and clinical information from roughly one-third of the U.S. population. This study utilizes SEER data to assess whether CRT + IMT improves survival in mCRC.

Given the limited research on immunotherapy for mCRC, this analysis evaluates its impact on overall survival (OS) and cancer-specific survival (CSS) based on the data from the SEER database. Additionally, key prognostic factors were identified to guide clinical decisions and future trial designs.

## Materials and methods

### Data source and patient selection

The SEER database, a key source for cancer incidence and survival data, is updated annually and publicly accessible. Data from 2000 to 2020 were collected using SEER*Stat (version 8.4.3). As SEER data are anonymized, this study did not require ethics approval or informed consent, following the Declaration of Helsinki guidelines. Patients with CRC diagnosed from 2010 to 2020 were screened using ICD-O-3 and AJCC TNM staging criteria. With pembrolizumab and nivolumab approved for MSI-H/dMMR mCRC in 2017, the study focused on patients diagnosed between 2018 and 2020 to assess immunotherapy effects, while patients from 2010 to 2015 served as the control cohort.

CRC cases were identified using ICD-O-3 codes (C18.0, C18.1, C18.2, C18.3, C18.4, C18.5, C18.6, C18.7, C19.9, and C20.9), excluding patients with other malignancies. Diagnostic periods were divided into 2010–2015 and 2018–2020. A total of 21 clinical variables were extracted from SEER, including patient ID, age, sex, race, marital status, tumor location, pathological grade, histological type, T stage, N stage, M stage, surgical information, specific sites of distant metastasis (encompassing bone, brain, liver, and lung), primary cause of death recorded by SEER, survival time, survival status, year of initial diagnosis, and sequence number. CRC-specific deaths were identified using SEER’s “death” code, while other causes were tracked by non-cancer death codes. The SEER staging system consists of four distinct stages: “*in situ*,” “localized,” “regional,” and “distant disease.” In this study, metastasis referred to tumors with distant metastases (T1-4 N0-2 M1).

#### Inclusion criteria

1. Patients who received both radiotherapy and chemotherapy.
2. Individuals with a primary colorectal tumor.
3. Patients aged 18 or older.

#### Exclusion criteria

1. Patients with multiple primary tumors.
2. Those with M0 or unknown metastatic status.
3. Patients with missing key clinical data (e.g., race, marital status, surgery details, pathological grade).
4. Cases with incomplete AJCC staging (T0, Tx, Nx) or missing data on metastasis to bone, brain, liver, or lung.
5. Individuals with uncertain survival duration or status.
6. Patients who did not survive beyond the first day.

**Figure 1:**
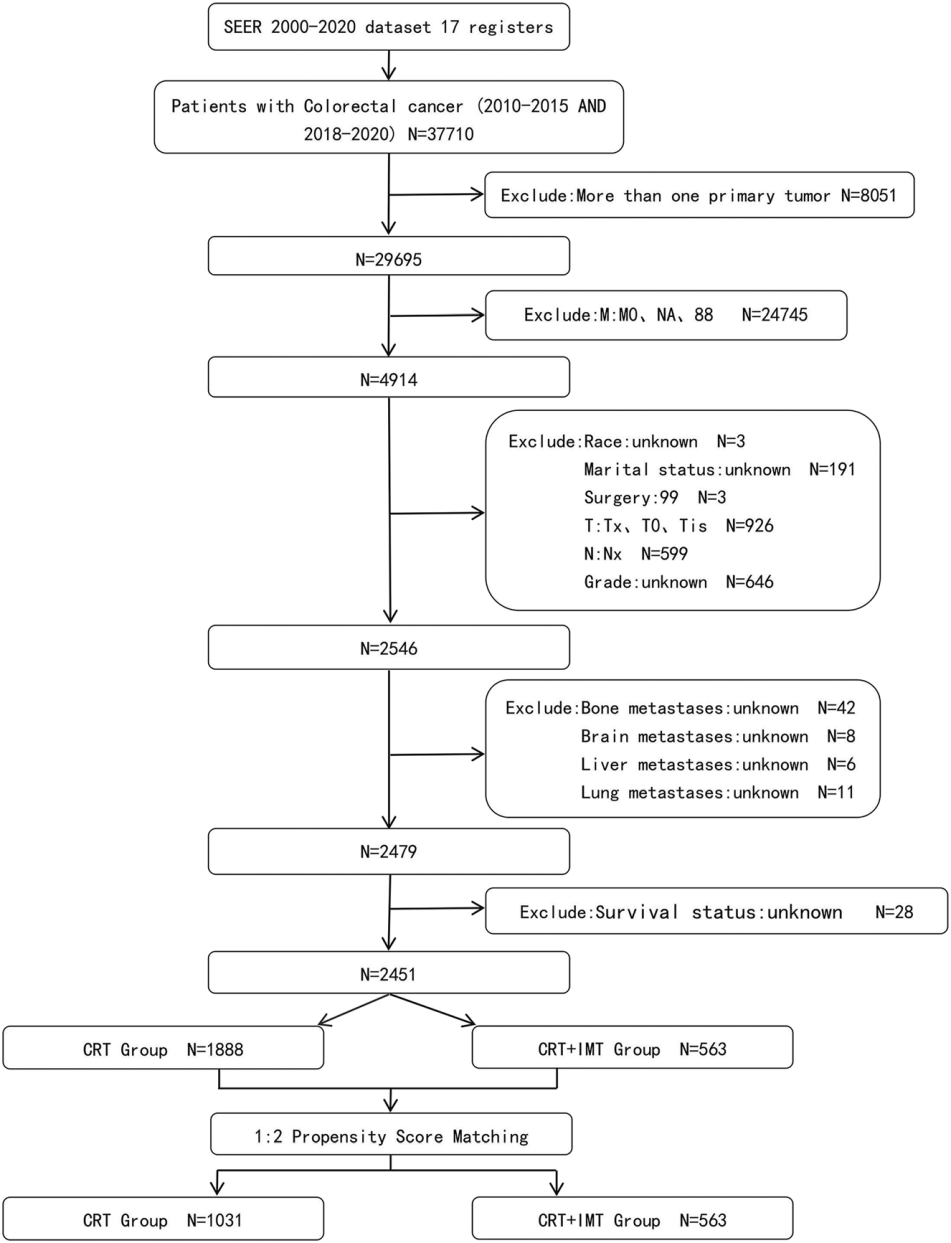
Flow diagram for the process of patient screening. CRT, chemoradiotherapy; IMT, immunotherapy.

The study population selection process is depicted in Figure 1.

### Study results

SEER data on mCRC were divided into two groups: CRT and CRT + IMT, based on the approval of immunotherapy as a treatment for mCRC.

The primary outcomes were OS and CSS. CSS was measured as the time from diagnosis to death from CRC. If the patient did not die from the disease or any cancer-related cause, it extended to the last recorded follow-up, while OS tracked the time from diagnosis to death from any cause or last follow-up. The cause of death classification in SEER was leveraged to determine the cause of death, with survival status noted as ‘alive,’ and survival time recorded in months.

### Statistical analysis

The χ^2^ test was leveraged to compare clinical characteristics between the “CRT + IMT” and “CRT” groups. To adjust for baseline differences and reduce selection bias, 1:2 propensity score matching (PSM) was executed with a caliper width of 0.1. A uniform 30-month cutoff was adopted for survival analysis to enable standardized comparison and visualization. The actual follow-up duration was sufficient to support the reported estimates of median survival.

Kaplan-Meier curves for OS and CSS were plotted using log-rank tests before and after PSM. A robust multivariable prognosis model was developed using a two-step variable selection approach. First, a univariable Cox regression analysis was performed on the matched cohort to identify candidate variables associated with survival outcomes. Second, to prevent overfitting and address multicollinearity among these candidates, feature selection was conducted using the least absolute shrinkage and selection operator (LASSO) regression with 10-fold cross-validation. The optimal penalization parameter (λ) was selected using the ‘one standard error’ (1-SE) rule to favor a more parsimonious model. All candidate variables from the univariable analysis, including the primary treatment variable (CRT + IMT vs. CRT), were incorporated into the LASSO analysis, and only those with non-zero coefficients at the optimal λ were retained for the final multivariable analysis. The treatment variable (CRT + IMT vs. CRT) was retained in the final model *a priori*, given its central role in testing the study hypothesis. Hazard ratios (HRs) alongside their 95 % confidence intervals (CIs) were calculated from the final multivariable Cox models based on the LASSO-selected variables.

Subgroup analyses were carried out to examine the robustness of the treatment relationships within subgroups and to delve into potential interactions between treatment and other variables. All tests were two-tailed, with significance set at p<0.05. R version 4.3.2 (R Foundation for Statistical Computing, Vienna, Austria) was employed for statistical analyses, with the MatchIt package for PSM and the glmnet package for LASSO regression.

### Ethical approval & informed consent

This study was implemented using data from the publicly available database, SEER, which contains de-identified information. Consequently, ethical approval and the need for informed consent were deemed unnecessary.

## Results

### Study cohort selection and PSM

A total of 2,451 mCRC patients were identified from SEER: 1,888 in the CRT group and 563 in the CRT + IMT group. The cohort was predominantly male (60.1 %), with a male-to-female ratio of 1.51:1. Among them, 60.1 % of patients were aged 60 or younger, and 78.3 % were White. Rectal cancer comprised 81.3 % of cases, with liver metastases being the most common (63.9 %). Significant baseline differences were found between the groups in race, histological type, pathological grade, surgery, T stage, and brain metastasis. After 1:2 PSM, the final matched cohort included 563 patients in the CRT + IMT group and 1,031 in the CRT group, with balanced characteristics (p>0.05, Table 1).

**Table 1:** Baseline characteristics of patients before and after PSM.

Variables	Originaldata set (n=2,451)			PSM data set (n=1,594)		
	CRT	CRT + IMT	p-Value	CRT	CRT + IMT	p-Value
	N=1,888	N=563		N=1,031	N=563	
Sex			0.242			0.337
Male	1,123 (59.5 %)	351 (62.3 %)		616 (59.7 %)	351 (62.3 %)	
Female	765 (40.5 %)	212 (37.7 %)		415 (40.3 %)	212 (37.7 %)	
Age			0.562			0.928
≤60	1,129 (59.8 %)	345 (61.3 %)		628 (60.9 %)	345 (61.3 %)	
>60	759 (40.2 %)	218 (38.7 %)		403 (39.1 %)	218 (38.7 %)	
Race			0.02			0.490
White	1,477 (78.2 %)	443 (78.7 %)		833 (80.8 %)	443 (78.7 %)	
Black	207 (11.0 %)	43 (7.6 %)		78 (7.6 %)	43 (7.6 %)	
Other	204 (10.8 %)	77 (13.7 %)		120 (11.6 %)	77 (13.7 %)	
Marital			0.729			0.704
Single	413 (21.9 %)	126 (22.4 %)		213 (20.7 %)	126 (22.4 %)	
Married/unmarried or domestic partner	1,108 (58.7 %)	336 (59.7 %)		624 (60.5 %)	336 (59.7 %)	
Separated/divorced/widowed	367 (19.4 %)	101 (17.9 %)		194 (18.8 %)	101 (17.9 %)	
Primary.site			0.317			0.728
Proximal colon	175 (9.3 %)	41 (7.3 %)		75 (7.3 %)	41 (7.3 %)	
Distal colon	188 (10.0 %)	54 (9.6 %)		112 (10.9 %)	54 (9.6 %)	
Rectum	1,525 (80.8 %)	468 (83.1 %)		844 (81.9 %)	468 (83.1 %)	
Histology			<0.001			0.453
Adenocarcinoma	1,522 (80.6 %)	533 (94.7 %)		965 (93.6 %)	533 (94.7 %)	
Other	366 (19.4 %)	30 (5.3 %)		66 (6.4 %)	30 (5.3 %)	
Grade			<0.001			0.313
I-II	1,424 (75.4 %)	488 (86.7 %)		873 (84.7 %)	488 (86.7 %)	
III-IV	464 (24.6 %)	75 (13.3 %)		158 (15.3 %)	75 (13.3 %)	
Surg			<0.001			0.859
No	732 (38.8 %)	108 (19.2 %)		203 (19.7 %)	108 (19.2 %)	
Yes	1,156 (61.2 %)	455 (80.8 %)		828 (80.3 %)	455 (80.8 %)	
T			0.021			0.359
T1	189 (10.0 %)	36 (6.4 %)		68 (6.6 %)	36 (6.4 %)	
T2	87 (4.6 %)	32 (5.7 %)		43 (4.2 %)	32 (5.7 %)	
T3	1,115 (59.1 %)	325 (57.7 %)		632 (61.3 %)	325 (57.7 %)	
T4	497 (26.3 %)	170 (30.2 %)		288 (27.9 %)	170 (30.2 %)	
N			0.257			0.640
N0	537 (28.4 %)	179 (31.8 %)		344 (33.4 %)	179 (31.8 %)	
N1	928 (49.2 %)	270 (48.0 %)		469 (45.5 %)	270 (48.0 %)	
N2	423 (22.4 %)	114 (20.2 %)		218 (21.1 %)	114 (20.2 %)	
Bone metastasis			0.079			0.814
No	1706 (90.4 %)	523 (92.9 %)		953 (92.4 %)	523 (92.9 %)	
Yes	182 (9.6 %)	40 (7.1 %)		78 (7.6 %)	40 (7.1 %)	
Brain metastasis			0.04			0.876
No	1824 (96.6 %)	554 (98.4 %)		1,012 (98.2 %)	554 (98.4 %)	
Yes	64 (3.4 %)	9 (1.6 %)		19 (1.8 %)	9 (1.6 %)	
Liver metastasis			0.649			0.841
No	686 (36.3 %)	198 (35.2 %)		356 (34.5 %)	198 (35.2 %)	
Yes	1,202 (63.7 %)	365 (64.8 %)		675 (65.5 %)	365 (64.8 %)	
Lung metastasis			0.056			0.913
No	1,370 (72.6 %)	432 (76.7 %)		795 (77.1 %)	432 (76.7 %)	
Yes	518 (27.4 %)	131 (23.3 %)		236 (22.9 %)	131 (23.3 %)	

### Survival results before and after PSM

**Figure 2:**
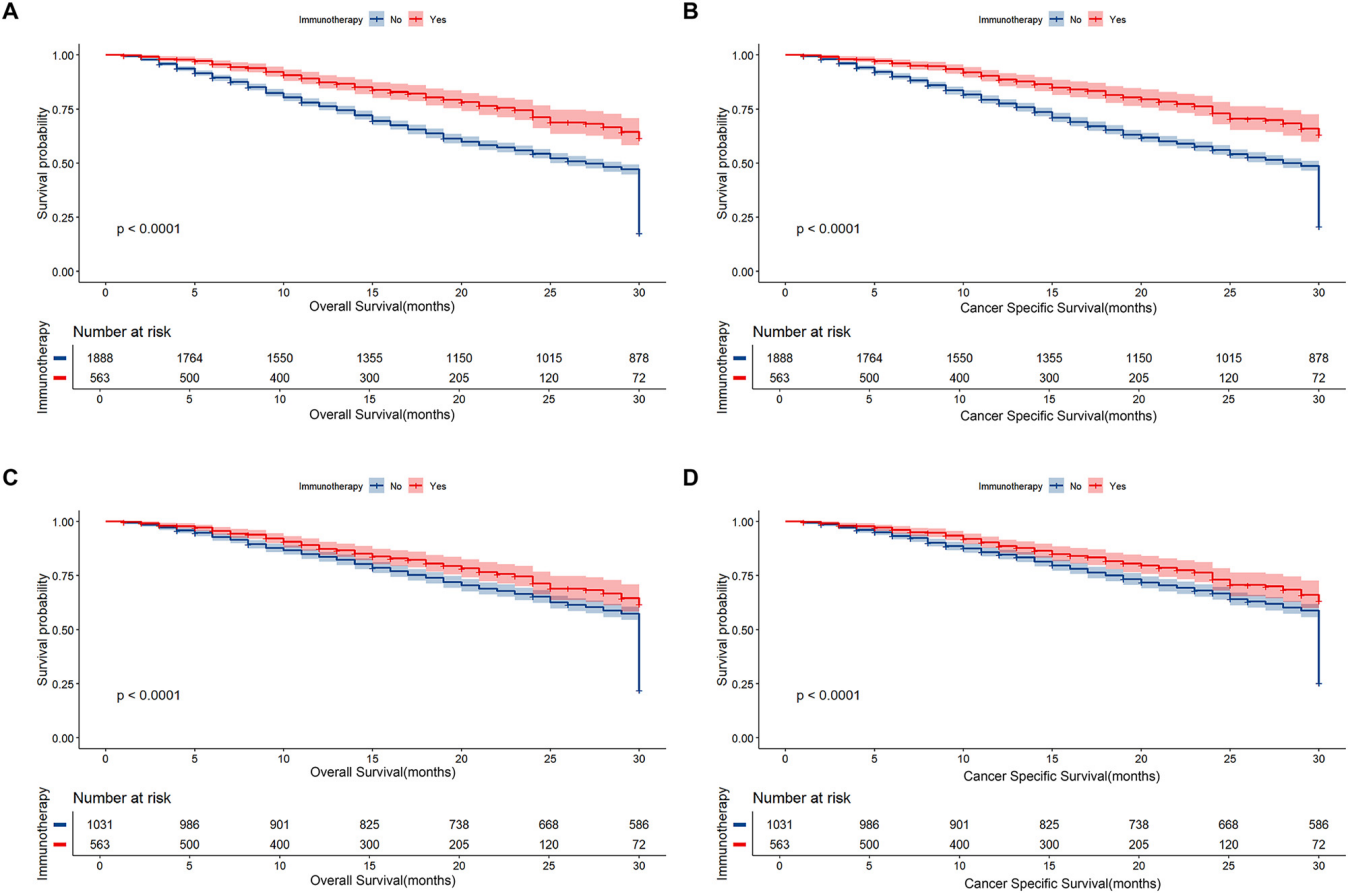
Kaplan-Meier curves for OS (A, C) and CSS (B, D) of patients before and after PSM. A 95 % confidence interval (estimated from a log hazard), the number of patients at risk at different time points, and the p value for the log-rank test are displayed on the graph.

Kaplan-Meier curves were leveraged to compute OS and CSS for the CRT and CRT + IMT groups. Before matching, the median OS was 29 months (95 % CI 28-30), and CSS was 30 months (95 % CI 29-30) for the entire cohort. The CRT + IMT group showed superior outcomes, with median OS and CSS not yet reached, while the CRT group had a median OS of 27 months (95 % CI 26-29) and CSS of 28 months (95 % CI 27-30). After matching, the median OS elevated to 30 months (95 % CI 30-30) and CSS to 30 months (95 % CI 30-30). In the CRT + IMT group, the median OS and CSS remained undetermined, while the CRT group had a median OS of 30 months (95 % CI 30-30) and CSS of 30 months (95 % CI 30-30, Table 2). Kaplan-Meier analysis revealed significantly prolonged OS (p<0.0001, Figure 2A) and CSS (p<0.0001, Figure 2B) in the CRT + IMT group. After matching, CRT + IMT considerably prolonged both OS (p<0.0001, Figure 2C) and CSS (p<0.0001, Figure 2D).

**Table 2:** Survival rate of patients stratified by treatment method.

**Variables**	**Originaldata set (n=2,451)**				**PSM data set (n=1,594)**			

	**All patients**	**CRT**	**CRT + IMT**	**p-Value**	**All patients**	**CRT**	**CRT + IMT**	**p-Value**
		**N=1,888**	**N=563**			**N=1,031**	**N=563**	
OS				p<0.0001				p<0.0001

6-month OS rate (95 %CI)	90.7 % (89.5–91.9 %)	89.3 % (88.0–90.7 %)	95.6 % (93.8–97.4 %)		93.6 % (92.4–94.8 %)	92.6 % (91.0–94.2 %)	95.6 % (93.8–97.4 %)	
12-month OS rate (95 %CI)	78.5 % (76.9–80.2 %)	76.3 % (74.4–78.3 %)	87.3 % (84.3–90.4 %)		84.7 % (82.9–86.6 %)	83.5 % (81.2–85.8 %)	87.3 % (84.3–90.4 %)	
18-month OS rate (95 %CI)	66.7 % (64.8–68.7 %)	63.7 % (61.5–65.9 %)	80.5 % (76.7–84.5 %)		75.8 % (73.6–78.0 %)	73.8 % (71.2–76.6 %)	80.5 % (76.7–84.5 %)	
24-month OS rate (95 %CI)	57.2 % (55.2–59.3 %)	54.3 % (52.1–56.6 %)	71.3 % (66.3–76.6 %)		67.0 % (64.6–69.6 %)	65.2 % (62.3–68.2 %)	71.3 % (66.3–76.6 %)	
30-month OS rate (95 %CI)	21.0 % (19.2–22.8 %)	17.7 % (16.1–19.5 %)	61.6 % (55.3–68.7 %)		26.4 % (24.0–29.1 %)	22.0 % (19.6–24.7 %)	61.6 % (55.3–68.7 %)	
Median OS (months)	29 (28–30)	27 (26–29)	NA		30 (30–30)	30 (30–30)	NA	

CSS				p<0.0001				p<0.0001

6-month CSS rate (95 %CI)	91.3 % (90.1–92.4 %)	89.9 % (88.6–91.3 %)	96.0 % (94.3–97.7 %)		94.1 % (93.0–95.3 %)	93.2 % (91.7–94.7 %)	96.0 % (94.3–97.7 %)	
12-month CSS rate (95 %CI)	79.9 % (78.3–81.5 %)	77.7 % (75.8–79.6 %)	88.5 % (85.6–91.5 %)		85.9 % (84.1–87.6 %)	84.6 % (82.4–86.8 %)	88.5 % (85.6–91.5 %)	
18-month CSS rate (95 %CI)	68.4 % (66.5–70.3 %)	65.4 % (63.3–67.6 %)	81.6 % (77.8–85.5 %)		76.9 % (74.7–79.1 %)	75.0 % (72.4–77.7 %)	81.6 % (77.8–85.5 %)	
24-month CSS rate (95 %CI)	59.0 % (57.0–61.1 %)	56.0 % (53.8–58.4 %)	73.0 % (68.0–78.3 %)		68.5 % (66.1–71.1 %)	66.6 % (63.8–69.6 %)	73.0 % (68.0–78.3 %)	
30-month CSS rate (95 %CI)	23.9 % (22.1–25.9 %)	20.7 % (18.9–22.6 %)	63.1 % (56.7–70.2 %)		29.5 % (27.0–32.3 %)	25.2 % (22.7–28.1 %)	63.1 % (56.7–70.2 %)	
Median CSS (months)	30 (29–30)	28 (27–30)	NA		30 (30–30)	30 (30–30)	NA	

### Cox regression analysis of survival

Based on the matched cohort, clinical variables associated with OS and CSS (p<0.05; Tables 3 and 4) were initially identified using univariable Cox regression analysis. To optimize variable selection for the multivariable model and mitigate the risk of overfitting, features were subsequently screened using LASSO regression (Figures S1 and S2). For OS, the following core prognostic variables were selected by LASSO regression: treatment modality (CRT + IMT vs. CRT alone), age, marital status, location of primary tumors, histologic grade, surgical status, T stage, N stage, bone metastasis, brain metastasis, and lung metastasis. For CSS, the variables selected included treatment modality, age, location of primary tumors, histologic grade, surgical status, T stage, N stage, bone metastasis, brain metastasis, and lung metastasis. CRT + IMT prolonged OS (HR=0.54, 95 % CI: 0.44–0.65) and CSS (HR=0.54, 95 % CI: 0.44–0.66) (Tables S1 and S2). Variables selected via LASSO regression were then incorporated into a multivariable Cox proportional hazards model. The results demonstrated that CRT + IMT was an independent protective factor for both OS (HR=0.54, 95 % CI: 0.44–0.65, p<0.001) and CSS (HR=0.54, 95 % CI: 0.44–0.66, p<0.001). In addition to the treatment effect, the multivariable analysis identified age >60 years, higher histologic grade (Ⅲ–Ⅳ), N2 stage, and the presence of bone, brain, and lung metastases as independent risk factors for poorer survival outcomes (Tables 3 and 4). Distal colon or rectal tumors, implementation of surgical treatment, and earlier T stage were associated with prolonged OS and CSS. After adjustment for this entire set of reliable covariates, the benefit of CRT + IMT remained consistent in both the OS model and the CSS model, highlighting the pronounced survival advantages conferred by adding immunotherapy to chemoradiotherapy.

**Table 3:** Univariable and multivariable Cox regression analyses for OS of patients after PSM.

Variables	N	Event N	Univariable			Multivariable		
			HR	95 %CI	p-Value	HR	95 %CI	p-Value
Sex								
Male	967	561	–	–				
Female	627	354	0.92	0.81, 1.05	0.240			
Age								
≤60	973	514	–	–		–	–	
>60	621	401	1.53	1.34, 1.75	<0.001	1.52	1.32, 1.74	<0.001
Race								
White	1,276	738	–	–				
Black	121	74	1.24	0.98, 1.58	0.073			
Other	197	103	0.88	0.72, 1.09	0.244			
Marital								
Single	339	198	–	–				
Married/unmarried or domestic partner	960	525	0.85	0.72, 1.00	0.047	0.81	0.69, 0.96	0.013
Separated/divorced/widowed	295	192	1.21	0.99, 1.48	0.056	0.94	0.76, 1.15	0.5
Primary.site								
Proximal colon	116	81	–	–		–	–	
Distal colon	166	110	0.58	0.43, 0.77	<0.001	0.63	0.47, 0.85	0.002
Rectum	1,312	724	0.46	0.37, 0.58	<0.001	0.49	0.38, 0.63	<0.001
Histology								
Adenocarcinoma	1,498	857	–	–				
Other	96	58	1.19	0.91, 1.55	0.201			
Grade								
I-II	1,361	757	–	–		–	–	
III-IV	233	158	1.60	1.35, 1.90	<0.001	1.60	1.35, 1.91	<0.001
Surg								
No	311	234	–	–		–	–	
Yes	1,283	681	0.38	0.33, 0.45	<0.001	0.38	0.32, 0.45	<0.001
T								
T1	104	72	–	–		–	–	
T2	75	36	0.46	0.31, 0.68	<0.001	0.65	0.43, 0.98	0.039
T3	957	520	0.53	0.41, 0.67	<0.001	0.76	0.58, 1.00	0.051
T4	458	287	0.82	0.63, 1.06	0.137	0.99	0.75, 1.31	>0.9
N								
N0	523	311	–	–		–	–	
N1	739	396	0.79	0.68, 0.91	0.002	1.10	0.94, 1.29	0.2
N2	332	208	1.09	0.91, 1.30	0.341	1.51	1.24, 1.83	<0.001
Bone metastasis								
No	1,476	822	–	–		–	–	
Yes	118	93	2.82	2.27, 3.50	<0.001	1.71	1.36, 2.15	<0.001
Brain metastasis								
No	1,566	896	–	–		–	–	
Yes	28	19	2.42	1.54, 3.81	<0.001	1.78	1.11, 2.86	0.017
Liver metastasis								
No	554	308	–	–				
Yes	1,040	607	1.10	0.96, 1.26	0.192			
Lung metastasis								
No	1,227	682	–	–		–	–	
Yes	367	233	1.33	1.15, 1.54	<0.001	1.34	1.15, 1.57	<0.001
Treat								
CRT	1,031	800	–	–		–	–	
CRT + IMT	563	115	0.52	0.42, 0.63	<0.001	0.54	0.44, 0.65	<0.001

**Table 4:** Univariable and multivariable Cox regression analyses for CSS of patients after PSM.

Variables	N	Event N	Univariable			Multivariable		
			HR	95 %CI	p-Value	HR	95 %CI	p-Value
Sex								
Male	967	525	–	–				
Female	627	335	0.94	0.82, 1.07	0.338			
Age								
≤60	973	489	–	–		–	–	
>60	621	371	1.47	1.28, 1.68	<0.001	1.46	1.27, 1.67	<0.001
Race								
White	1,276	698	–	–				
Black	121	65	1.15	0.89, 1.48	0.282			
Other	197	97	0.88	0.71, 1.09	0.236			
Marital								
Single	339	187	–	–				
Married/unmarried or domestic partner	960	502	0.86	0.73, 1.02	0.085			
Separated/divorced/widowed	295	171	1.12	0.91, 1.38	0.285			
Primary.site								
Proximal colon	116	78	–	–		–	–	
Distal colon	166	103	0.55	0.41, 0.73	<0.001	0.60	0.44, 0.81	<0.001
Rectum	1,312	679	0.44	0.35, 0.56	<0.001	0.47	0.37, 0.61	<0.001
Histology								
Adenocarcinoma	1,498	802	–	–				
Other	96	58	1.29	0.99, 1.69	0.060			
Grade								
I-II	1,361	712	–	–		–	–	
III-IV	233	148	1.61	1.35, 1.92	<0.001	1.60	1.33, 1.91	<0.001
Surg								
No	311	222	–	–		–	–	
Yes	1,283	638	0.38	0.33, 0.44	<0.001	0.37	0.31, 0.44	<0.001
T								
T1	104	69	–	–		–	–	
T2	75	34	0.45	0.30, 0.68	<0.001	0.64	0.42, 0.97	0.036
T3	957	483	0.51	0.39, 0.65	<0.001	0.72	0.55, 0.95	0.019
T4	458	274	0.82	0.63, 1.07	0.136	0.97	0.73, 1.29	0.8
N								
N0	523	289	–	–		–	–	
N1	739	374	0.81	0.69, 0.94	0.006	1.15	0.98, 1.36	0.095
N2	332	197	1.12	0.93, 1.34	0.231	1.59	1.31, 1.94	<0.001
Bone metastasis								
No	1,476	772	–	–				
Yes	118	88	2.75	2.20, 3.43	<0.001	1.68	1.32, 2.13	<0.001
Brain metastasis								
No	1,566	841	–	–				
Yes	28	19	2.62	1.66, 4.13	<0.001	1.84	1.15, 2.96	0.012
Liver metastasis								
No	554	280	–	–		–	–	
Yes	1,040	580	1.16	1.01, 1.34	0.041			
Lung metastasis								
No	1,227	639	–	–		–	–	
Yes	367	221	1.34	1.15, 1.56	<0.001	1.37	1.17, 1.61	<0.001
Treat								
CRT	1,031	753	–	–		–	–	
CRT + IMT	563	107	0.52	0.42, 0.64	<0.001	0.54	0.44, 0.66	<0.001

### Subgroup analysis after PSM

**Figure 3:**
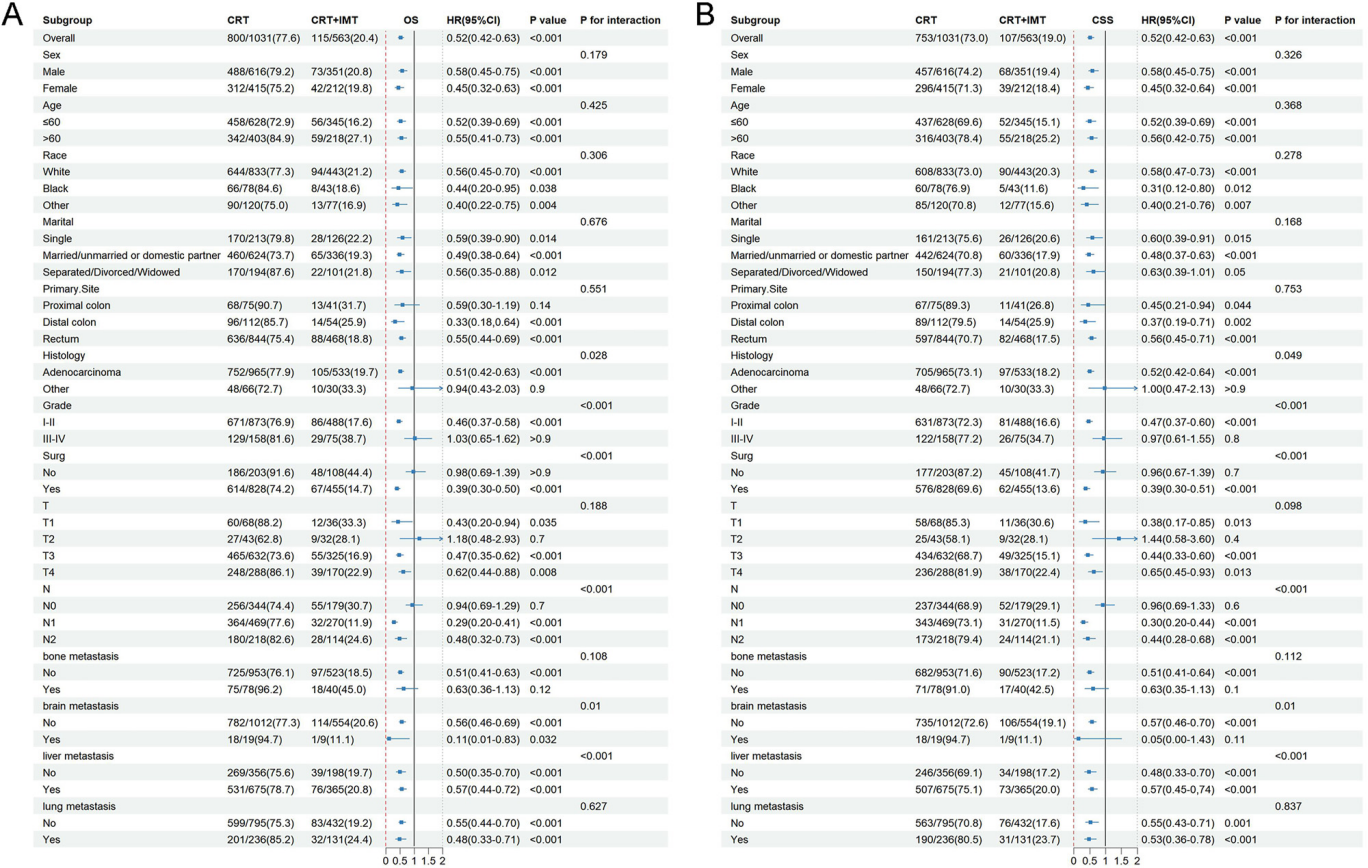
Results for the subgroup analyses and interaction tests of treatment-based effects on OS (A) and CSS (B) are summarized in a forest plot.

The subgroup analyses and the interaction assessment were conducted to clarify the impact of CRT + IMT on survival among patients suffering from mCRC (Figure 3). In this analysis, multivariable Cox regression within each subgroup was adjusted for the core prognostic variables previously selected by LASSO regression (OS model: treatment modality [CRT + IMT vs. CRT alone], age, marital status, location of primary tumors, histologic grade, surgical status, T stage, N stage, and presence of bone, brain, and lung metastases; CSS model: treatment modality, age, location of primary tumors, histologic grade, surgical status, T stage, N stage, and presence of bone, brain, and lung metastases). The subgroup analysis indicated that CRT + IMT significantly prolonged both OS and CSS. In the overall population, CRT + IMT significantly extended OS (HR=0.52, 95 % CI: 0.42–0.63, p<0.001) and CSS (HR=0.52, 95 % CI: 0.42–0.63, p<0.001) compared with CRT alone. This suggested that CRT + IMT not only prolonged OS but also specifically reduced the risk of cancer-specific mortality, providing dual solid evidence for the clinical benefits of CRT + IMT. The subgroup analyses further revealed that benefits from CRT + IMT were observed regardless of sex, age, race, marital status, or status of lung metastasis, with no significant interactions detected for these factors (p for interaction >0.05). Particularly pronounced benefits from CRT + IMT were noted among patients with grade Ⅰ-Ⅱ tumors (OS: HR=0.46, p<0.001; CSS: HR=0.47, p<0.001), those who underwent surgery (OS: HR=0.39, p<0.001; CSS: HR=0.39, p<0.001), those with lymph node metastasis (N1 stage: OS HR=0.29, p<0.001, CSS HR=0.30, p<0.001; N2 stage: OS HR=0.48, p<0.001, CSS HR=0.44, p<0.001), those with adenocarcinoma (OS: HR=0.51, p<0.001; CSS: HR=0.52, p<0.001), those with T3 or T4 stage tumors (T3: OS HR=0.47, p<0.001, CSS HR=0.44, p<0.001; T4: OS HR=0.62, p=0.008, CSS HR=0.65, p=0.013), those with distal colon cancer (OS: HR=0.33, p<0.001; CSS: HR=0.37, p=0.002), those without bone metastasis (OS: HR=0.51, p<0.001; CSS: HR=0.51, p<0.001), and those without brain metastasis (OS: HR=0.56, p<0.001; CSS: HR=0.57, p<0.001). In contrast, no significant survival benefit was observed among individuals with proximal colon cancer, those with histologic grade Ⅲ-Ⅳ, those who did not undergo surgery, or those without lymph node metastasis (N0). The interaction analysis indicated that histologic grade, surgical status, N stage, and the presence of liver or brain metastasis significantly influenced the therapeutic effects on both OS and CSS (p for interaction <0.05). Among these, patients with lower-grade tumors, those who underwent surgery, those with lymph node metastasis, or those without brain metastasis benefited more from CRT + IMT.

## Discussion

Immunotherapy has emerged as the fourth pillar of cancer treatment, alongside surgery, chemotherapy, and radiotherapy, and is increasingly essential in combination therapies. Our study corroborates the effectiveness of CRT + IMT in mCRC, particularly in specific subgroups. Landmark trials like GERCOR NIPICOL, KEYNOTE-016, KEYNOTE-164, CheckMate-142, and KEYNOTE-177 have shown immunotherapy’s success in treating unresectable or metastatic dMMR/MSI-H CRC.

While dMMR/MSI-H patients benefit significantly from immunotherapy, they represent only 5 % of mCRC cases. The use of ICIs alone in pMMR/MSS patients (95 % of cases) has been less efficacious due to the immunosuppressive nature of pMMR/MSS tumors, which have low tumor-infiltrating lymphocytes and low tumor mutation burden (TMB). Overcoming these challenges by turning cold tumors into hot tumors is crucial [26]. Combining ICIs with other therapies could improve outcomes for pMMR/MSS patients by altering the tumor microenvironment and may also enhance immunotherapy efficacy in dMMR/MSI-H patients by increasing TMB [27].

Our study, based on SEER data, explored the synergistic effect of CRT + IMT in mCRC. The results unveiled a considerable survival advantage of this combined approach for patients with mCRC, offering promising insights into the treatment of mCRC, especially pMMR/MSS, for which single-agent immunotherapy has traditionally been less effective.

We analyzed 2,451 mCRC patients diagnosed between 2010 and 2020. To date, this represents one of the most comprehensive studies exploring CRT + IMT in mCRC treatment.

Theoretically, there is a synergy between chemotherapy and immunotherapy. Tumor cells attacked by chemotherapy drugs are exposed to antigens, triggering an immune response and reducing the tumor’s ability to evade the immune system [28]. Despite this promising mechanism, clinical outcomes in pMMR/MSS patients treated with immunotherapy alone have generally been modest. For instance, the METIMMOX study, which assessed the impact of combining chemotherapy (FLOX regimen) with nivolumab, demonstrated a median PFS of 6.6 months in the combination group compared to 5.6 months in the chemotherapy-only group; the results were not particularly encouraging [29]. Radiotherapy promotes immunogenic cell death by enhancing T-cell responses and releasing tumor-associated antigens, which attract immune cells and modify the tumor microenvironment, boosting antitumor activity [30], 31]. The prior VOLTAGE study unraveled a 30 % pathological complete response (pCR) in MSS patients and 60 % in microsatellite instability-high (MSI-H) patients after preoperative CRT plus nivolumab [32]. Similarly, the AVANA phase II trial (NCT03854799) reported a 23 % pCR in patients treated with CRT plus avelumab, among whom 61.5 % experienced severe pathological responses [33]. These findings highlight the potential of CRT + IMT to improve outcomes in mCRC. Although our study did not explore the mechanisms behind this combination, prior research unraveled several explanations. First, radiotherapy or CRT induces immunogenic cell death (ICD) [34], while chemotherapy releases damage-associated molecular patterns (DAMPs) and tumor neoantigens to trigger immune responses [35]. Second, CRT directly reduces tumor size through cell destruction. Due to reduced tumor size and volume, the immune system can more effectively identify and destroy cancer cells, which enhances the effectiveness of immunotherapy [36]. This effect is most prominent during the initial phases of treatment before tumors develop sophisticated mechanisms to evade immune detection. Third, radiotherapy can result in a systemic immune response known as the abscopal effect, where local treatment leads to the suppression of distant, untreated tumors [37]. This phenomenon holds promise for improving outcomes in mCRC and could complement current immunotherapy strategies. Fourth, radiotherapy affects the tumor microenvironment by shifting it from an immunosuppressive to an immunostimulatory state. It can increase PD-L1 expression on tumor cells [38]. Upon, radiotherapy, tumors release double-stranded DNA, activating the cGAS-STING pathway, which upregulates PD-L1 and thus induce tumor immune escape [39]. Furthermore, hypoxia in tumors, often due to irregular or impaired blood vessels, can contribute to tumor resistance to radiotherapy. ICIs have been demonstrated to correct these abnormal blood vessels, decreasing hypoxia and making tumors more responsive to radiotherapy. This improvement in tumor microenvironment boosts the overall effectiveness of CRT + IMT [40].

Moreover, the research by Lieu et al. has unveiled that older age (over 65) is associated with poorer survival outcomes when age is deemed as a continuous variable [41]. This could be attributed to shorter life expectancy, greater comorbidities, or a reduced ability to tolerate aggressive chemotherapy in the old population [42], while younger patients generally exhibit better physical resilience [43]. Increased use of screening methods like fecal tests, endoscopy, and CT colonography has reduced CRC incidence in adults aged over 50, while the incidence rates are rising in younger adults [44]. In our study, 60.1 % of mCRC cases occurred in patients aged 60 or younger. Multivariable Cox regression analysis unveiled that patients aged over 60 had significantly worse OS and CSS compared to those aged 60 or younger. This finding underscores the critical role of age in survival outcomes. It is imperative to formulate age-specific treatment strategies to improve care for older mCRC patients.

Our research classified tumors by location: right-sided (proximal colon), left-sided (distal colon), and rectum. Survival outcomes were better for left-sided and rectal tumors compared to right-sided tumors, consistent with prior studies [45]. Right-sided colon cancers generally are linked to poorer survival because of the higher prevalence of BRAF mutations and increased microsatellite instability in these tumors [46], 47].

Beyond molecular differences related to location, some studies have indicated that different segments of the colon (encompassing the rectum) have distinct embryonic origins [48]. Additionally, variations in the gut microbiome, fecal composition, enzymes, and metabolites may lead to variations in the colon [49], 50]. Our findings suggest that left colon and rectal tumors are linked with more favorable OS and CSS outcomes.

Siebenhuner et al. analyzed SEER data from 2010 to 2015 on adults with mCRC between 2010 and 2015 who underwent both primary tumor resection and metastatic tumor resection. They unveiled a median OS of 31.2 months for patients who received both resections, compared to 20.4 months for those who did not [51]. European studies also support the survival benefit of resecting both tumor sites in mCRC patients [52], [53], [54], highlighting the linkage of surgical intervention with the overall prognosis of this population. Additionally, our subgroup analysis revealed that surgery combined with CRT + IMT further improved OS and CSS compared to individuals who did not undergo surgery.

Tumor differentiation is a crucial pathological factor. Poorly differentiated tumors are often correlated with worse clinical outcomes [55]. Poorly differentiated CRCs tend to exhibit increased bowel invasion, lymph node involvement, and vascular infiltration, suggesting that they are significant risk factors for disease progression [56]. Our findings align with previous research [57], 58], showing that poorly differentiated mCRC is linked to worse outcomes. Our subgroup analysis also revealed that CRT + IMT was more effective in improving OS and CSS in grades I-II patients compared to grades III-IV patients.

The findings of this study must be interpreted in light of limitations stemming from its retrospective design and the nature of the SEER database. First, as an observational analysis, it only revealed associations rather than established causality, in which case methods such as Mendelian randomization might be needed. Second, the SEER database lacks detailed clinical and molecular information. (ⅰ) Specific treatment details are not captured, including type of immunotherapy agent (e.g., anti-PD-1, anti-CTLA-4 antibodies), timing and sequence of treatment administration, chemoradiotherapy regimens, or targeted therapies. (ⅱ) Critically, key biomarker data, such as microsatellite instability (MSI) or mismatch repair (MMR) status, are unavailable, which are established predictive biomarkers for immunotherapy response in CRC. Consequently, the survival benefit observed for CRT + IMT in our analysis possibly reflects a heterogeneous effect and may be particularly driven by subgroups with specific tumor biology, such as MSI-H/dMMR status, which cannot be identified or accounted for in this dataset. (ⅲ) Only OS and CSS are provided, lacking other crucial efficacy endpoints such as PFS, objective response rate, disease control rate, and quality-of-life measures, thereby limiting a comprehensive assessment of treatment benefit. Third, the follow-up duration for immunotherapy-treated patients in SEER is relatively short, which may affect the reliability of long-term survival outcomes. Finally, although PSM was used to control for known confounders, unmeasured variables may still introduce residual bias. Prospective studies that include comprehensive molecular profiling and detailed treatment records are, therefore, necessary to validate these findings and to identify the patient subgroups most suitable for CRT + IMT.

The findings suggest promising directions for future investigation. First, conducting additional clinical trials is imperative to corroborate the effectiveness of CRT + IMT for mCRC. Second, further research should examine the impact of different immunotherapeutic agents, chemotherapy regimens, radiotherapy timing and dosage, fractionation patterns, and treatment duration on mCRC prognosis to identify optimal treatment strategies. Lastly, personalized treatment approaches are imperative for the management of mCRC. Given potential disparities in the efficacy of immunotherapy across different patients, identifying patients who may benefit most from immunotherapy can assist in formulating more targeted and effective treatment plans.

## Conclusions

Our study demonstrated that CRT + IMT significantly improves survival outcomes in mCRC patients compared to CRT alone. Key prognostic factors for OS and CSS included age, tumor location, pathological grade, surgery, T stage (T2, T3), N2 stage, metastases to bone, brain, and lungs, and treatment approaches. Our evidence corroborates the benefits of CRT + IMT, particularly in specific mCRC subgroups, furnishing further insights into the management of CRC. Nonetheless, larger prospective trials are desired to confirm these findings and inform future treatment strategies.

## Supplementary Material

Supplementary Material

## References

[1] Siegel RL, Wagle NS, Cercek A, Smith RA, Jemal A (2023). Colorectal cancer statistics, 2023. CA Cancer J Clin.

[2] Kanth P, Inadomi JM (2021). Screening and prevention of colorectal cancer. BMJ.

[3] Siegel RL, Miller KD, Fedewa SA, Ahnen DJ, Meester RG, Barzi A (2017). Colorectal cancer statistics, 2017. CA Cancer J Clin.

[4] Dekker E, Tanis PJ, Vleugels JL, Kasi PM, Wallace MB (2019). Colorectal cancer. Lancet (London, England).

[5] Biller LH, Schrag D (2021). Diagnosis and treatment of metastatic colorectal cancer: a review. JAMA.

[6] Underwood PW, Ruff SM, Pawlik TM (2024). Update on targeted therapy and immunotherapy for metastatic colorectal cancer. Cells.

[7] Rumpold H, Niedersüß-Beke D, Heiler C, Falch D, Wundsam HV, Metz-Gercek S (2020). Prediction of mortality in metastatic colorectal cancer in a real-life population: a multicenter explorative analysis. BMC Cancer.

[8] Stebbing J, Wasan HS (2019). Decoding metastatic colorectal cancer to improve clinical decision making.

[9] Benson AB, Venook AP, Adam M, Chang G, Chen Y-J, Ciombor KK (2024). Colon cancer, version 3.2024, NCCN clinical practice guidelines in oncology. J Natl Compr Cancer Netw.

[10] Chau I, Cunningham D (2009). Treatment in advanced colorectal cancer: what, when and how?. Br J Cancer.

[11] Le DT, Durham JN, Smith KN, Wang H, Bartlett BR, Aulakh LK (2017). Mismatch repair deficiency predicts response of solid tumors to PD-1 blockade. Science.

[12] Overman MJ, McDermott R, Leach JL, Lonardi S, Lenz H-J, Morse MA (2017). Nivolumab in patients with metastatic DNA mismatch repair-deficient or microsatellite instability-high colorectal cancer (CheckMate 142): an open-label, multicentre, phase 2 study. Lancet Oncol.

[13] Sun H, Shi K, Qi K, Kong H, He Q, Zhou M (2020). Pseudostellaria heterophylla extract polysaccharide H-1-2 suppresses pancreatic cancer by inhibiting hypoxia-induced AG2. Mol Ther Oncolytics.

[14] Le DT, Kim TW, Van Cutsem E, Geva R, Jäger D, Hara H (2020). Phase II open-label study of pembrolizumab in treatment-refractory, microsatellite instability–high/mismatch repair–deficient metastatic colorectal cancer: KEYNOTE-164. J Clin Oncol.

[15] Arrichiello G, Poliero L, Borrelli C, Paragliola F, Nacca V, Napolitano S (2021). Immunotherapy in colorectal cancer: is the long-awaited revolution finally happening?. Cancer Treat Res Commun.

[16] Leowattana W, Leowattana P, Leowattana T (2023). Systemic treatment for metastatic colorectal cancer. World J Gastroenterol.

[17] Watkinson R, Tam J, Vaysburd M, James L (2013). Simultaneous neutralization and innate immune detection of a replicating virus by TRIM21. J Virol.

[18] Wang W, Wu L, Zhang J, Wu H, Han E, Guo Q (2017). Chemoimmunotherapy by combining oxaliplatin with immune checkpoint blockades reduced tumor burden in colorectal cancer animal model. Biochem Biophys Res Commun.

[19] Kanterman J, Sade-Feldman M, Biton M, Ish-Shalom E, Lasry A, Goldshtein A (2014). Adverse immunoregulatory effects of 5FU and CPT11 chemotherapy on myeloid-derived suppressor cells and colorectal cancer outcomes. Cancer Res.

[20] Lote H, Starling N, Pihlak R, Gerlinger M (2022). Advances in immunotherapy for MMR proficient colorectal cancer. Cancer Treat Rev.

[21] Pfirschke C, Engblom C, Rickelt S, Cortez-Retamozo V, Garris C, Pucci F (2016). Immunogenic chemotherapy sensitizes tumors to checkpoint blockade therapy. Immunity.

[22] Herting CJ, Farren MR, Tong Y, Liu Z, O’Neil B, Bekaii-Saab T (2021). A multi-center, single-arm, phase Ib study of pembrolizumab (MK-3475) in combination with chemotherapy for patients with advanced colorectal cancer: HCRN GI14-186. Cancer Immunol Immunother.

[23] Antoniotti C, Rossini D, Pietrantonio F, Catteau A, Salvatore L, Lonardi S (2022). Upfront FOLFOXIRI plus bevacizumab with or without atezolizumab in the treatment of patients with metastatic colorectal cancer (AtezoTRIBE): a multicentre, open-label, randomised, controlled, phase 2 trial. Lancet Oncol.

[24] Parikh AR, Szabolcs A, Allen JN, Clark JW, Wo JY, Raabe M (2021). Radiation therapy enhances immunotherapy response in microsatellite stable colorectal and pancreatic adenocarcinoma in a phase II trial. Nat Cancer.

[25] Segal NH, Kemeny NE, Cercek A, Reidy DL, Raasch PJ, Warren P (2016). Non-randomized phase II study to assess the efficacy of pembrolizumab (Pem) plus radiotherapy (RT) or ablation in mismatch repair proficient (pMMR) metastatic colorectal cancer (mCRC) patients.

[26] Ganesh K, Stadler ZK, Cercek A, Mendelsohn RB, Shia J, Segal NH (2019). Immunotherapy in colorectal cancer: rationale, challenges and potential. Nat Rev Gastroenterol Hepatol.

[27] Borelli B, Antoniotti C, Carullo M, Germani MM, Conca V, Masi G (2022). Immune-checkpoint inhibitors (ICIs) in metastatic colorectal cancer (mCRC) patients beyond microsatellite instability. Cancers.

[28] Moretto R, Elliott A, Zhang J, Arai H, Germani MM, Conca V (2022). Homologous recombination deficiency alterations in colorectal cancer: clinical, molecular, and prognostic implications. JNCI (J Natl Cancer Inst).

[29] Ree AH, Hamre H, Kersten C, Hofsli E, Guren MG, Sorbye H (2021). Repeat sequential oxaliplatin-based chemotherapy (FLOX) and nivolumab versus FLOX alone as first-line treatment of microsatellite-stable (MSS) metastatic colorectal cancer (mCRC): initial results from the randomized METIMMOX study.

[30] Xue M, Tian Y, Sui Y, Zhao H, Gao H, Liang H (2022). Protective effect of fucoidan against iron overload and ferroptosis-induced liver injury in rats exposed to alcohol. Biomed Pharmacother.

[31] Voronova V, Vislobokova A, Mutig K, Samsonov M, Peskov K, Sekacheva M (2022). Combination of immune checkpoint inhibitors with radiation therapy in cancer: a hammer breaking the wall of resistance. Front Oncol.

[32] Yuki S, Bando H, Tsukada Y, Inamori K, Komatsu Y, Homma S (2020). Short-term results of VOLTAGE-A: nivolumab monotherapy and subsequent radical surgery following preoperative chemoradiotherapy in patients with microsatellite stable and microsatellite instability-high locally advanced rectal cancer.

[33] Salvatore L, Bensi M, Corallo S, Bergamo F, Pellegrini I, Rasola C (2021). Phase II study of preoperative (PREOP) chemoradiotherapy (CTRT) plus avelumab (AVE) in patients (PTS) with locally advanced rectal cancer (LARC): the AVANA study.

[34] Jin Y, Jiang J, Mao W, Bai M, Chen Q, Zhu J (2024). Treatment strategies and molecular mechanism of radiotherapy combined with immunotherapy in colorectal cancer. Cancer Lett.

[35] Krysko DV, Garg AD, Kaczmarek A, Krysko O, Agostinis P, Vandenabeele P (2012). Immunogenic cell death and DAMPs in cancer therapy. Nat Rev Cancer.

[36] Schumacher TN, Schreiber RD (2015). Neoantigens in cancer immunotherapy. Science.

[37] Mole R (1953). Whole body irradiation – radiobiology or medicine?. Br J Radiol.

[38] Deng L, Liang H, Burnette B, Beckett M, Darga T, Weichselbaum RR (2014). Irradiation and anti–PD-L1 treatment synergistically promote antitumor immunity in mice. J Clin Investig.

[39] Du S-S, Chen G-W, Yang P, Chen Y-X, Hu Y, Zhao Q-Q (2022). Radiation therapy promotes hepatocellular carcinoma immune cloaking via PD-L1 upregulation induced by cGAS-STING activation. Int J Radiat Oncol Biol Phys.

[40] Bagchi S, Yuan R, Engleman EG (2021). Immune checkpoint inhibitors for the treatment of cancer: clinical impact and mechanisms of response and resistance. Annu Rev Pathol.

[41] Lieu CH, Renfro LA, De Gramont A, Meyers JP, Maughan TS, Seymour MT (2014). Association of age with survival in patients with metastatic colorectal cancer: analysis from the ARCAD clinical trials program. J Clin Oncol.

[42] Bradley CJ, Yabroff KR, Warren JL, Zeruto C, Chawla N, Lamont EB (2016). Trends in the treatment of metastatic colon and rectal cancer in elderly patients. Med Care.

[43] Miller RA (1996). The aging immune system: primer and prospectus. Science.

[44] Murphy CC, Sandler RS, Sanoff HK, Yang YC, Lund JL, Baron JA (2017). Decrease in incidence of colorectal cancer among individuals 50 years or older after recommendations for population-based screening. Clin Gastroenterol Hepatol.

[45] Lv M-Y, Chen X-J, Chen J-G, Zhang B, Lin Y-Y, Huang T-Z (2022). Nomogram for predicting overall survival time of patients with stage IV colorectal cancer. Gastroenterol Rep.

[46] Ueda K, Yamada T, Ohta R, Matsuda A, Sonoda H, Kuriyama S (2022). BRAF V600E mutations in right-side colon cancer: heterogeneity detected by liquid biopsy. Eur J Surg Oncol.

[47] Papagiorgis PC, Zizi AE, Tseleni S, Oikonomakis IN, Nikiteas NI (2012). The pattern of epidermal growth factor receptor variation with disease progression and aggressiveness in colorectal cancer depends on tumor location. Oncol Lett.

[48] Koliaraki V, Pallangyo CK, Greten FR, Kollias G (2017). Mesenchymal cells in colon cancer. Gastroenterology.

[49] Xi Y, Yuefen P, Wei W, Quan Q, Jing Z, Jiamin X (2019). Analysis of prognosis, genome, microbiome, and microbial metabolome in different sites of colorectal cancer. J Transl Med.

[50] Zhong M, Xiong Y, Ye Z, Zhao J, Zhong L, Liu Y (2020). Microbial community profiling distinguishes left-sided and right-sided colon cancer. Front Cell Infect Microbiol.

[51] Siebenhüner AR, Güller U, Warschkow R (2020). Population-based SEER analysis of survival in colorectal cancer patients with or without resection of lung and liver metastases. BMC Cancer.

[52] Venderbosch S, de Wilt JH, Teerenstra S, Loosveld OJ, van Bochove A, Sinnige HA (2011). Prognostic value of resection of primary tumor in patients with stage IV colorectal cancer: retrospective analysis of two randomized studies and a review of the literature. Ann Surg Oncol.

[53] Van Rooijen K, Shi Q, Goey K, Meyers J, Heinemann V, Diaz-Rubio E (2018). Prognostic value of primary tumour resection in synchronous metastatic colorectal cancer: individual patient data analysis of first-line randomised trials from the ARCAD database. Eur J Cancer.

[54] Stelzner S, Radulova‐Mauersberger O, Zschuppe E, Kittner T, Abolmaali N, Puffer E (2019). Prognosis in patients with synchronous colorectal cancer metastases after complete resection of the primary tumor and the metastases. J Surg Oncol.

[55] Cooper H, Slemmer J (1991). Surgical pathology of carcinoma of the colon and rectum.

[56] Blumberg D, Paty PB, Picon AI, Guillem JG, Klimstra DS, Minsky BD (1998). Stage I rectal cancer: identification of high-risk patients. J Am Coll Surg.

[57] Ueno H, Konishi T, Ishikawa Y, Shimazaki H, Ueno M, Aosasa S (2015). Prognostic value of poorly differentiated clusters in the primary tumor in patients undergoing hepatectomy for colorectal liver metastasis. Surgery.

[58] Wang W, Yang Z-L, Liu J-Q, Yang L-P, Yang X-J, Fu X (2014). Overexpression of MTA1 and loss of KAI-1 and KiSS-1 expressions are associated with invasion, metastasis, and poor-prognosis of gallbladder adenocarcinoma. Tumori J.

