# Supplementary material for: Survival benefits of immunotherapy combined with chemoradiotherapy in metastatic colorectal cancer: an analysis based on data from the SEER database
Source: Open Med (Wars). 2026 Apr 27;21(1):20261416. doi: 10.1515/med-2026-1416 (PMC13124230; doi:10.1515/med-2026-1416)
Supplement: Supplementary file 1 — Supplementary Material [file j_med-2026-1416_suppl_001.docx]

**Supplementary Materials**

**Table S1** LASSO regression analysis for screening variables of overall survival in metastatic colorectal cancer patients

| Variables | Multivariable | | |
| --- | --- | --- | --- |
|  | HR | 95%CI | P-value |
| Age |  |  |  |
| ≤60 | - | - |  |
| ＞60 | 1.52 | 1.32,1.74 | <0.001 |
| Marital |  |  |  |
| Single | - | - |  |
| Married/unmarried or domestic partner | 0.81 | 0.69,0.96 | 0.013 |
| Separated/Divorced/Widowed | 0.94 | 0.76,1.15 | 0.5 |
| Primary.Site |  |  |  |
| Proximal colon | - | - |  |
| Distal colon | 0.63 | 0.47,0.85 | 0.002 |
| Rectum | 0.49 | 0.38,0.63 | <0.001 |
| Grade |  |  |  |
| I-II | - | - |  |
| III-IV | 1.60 | 1.35,1.91 | <0.001 |
| Surg |  |  |  |
| No | - | - |  |
| Yes | 0.38 | 0.32,0.45 | <0.001 |
| T |  |  |  |
| T1 | - | - |  |
| T2 | 0.65 | 0.43,0.98 | 0.039 |
| T3 | 0.76 | 0.58,1.00 | 0.051 |
| T4 | 0.99 | 0.75,1.31 | >0.9 |
| N |  |  |  |
| N0 | - | - |  |
| N1 | 1.10 | 0.94,1.29 | 0.2 |
| N2 | 1.51 | 1.24,1.83 | <0.001 |
| bone metastasis |  |  |  |
| No | - | - |  |
| Yes | 1.71 | 1.36,2.15 | <0.001 |
| brain metastasis |  |  |  |
| No | - | - |  |
| Yes | 1.78 | 1.11,2.86 | 0.017 |
| lung metastasis |  |  |  |
| No | - | - |  |
| Yes | 1.34 | 1.15,1.57 | <0.001 |
| Treat |  |  |  |
| CRT | - | - |  |
| CRT+IMT | 0.54 | 0.44,0.65 | <0.001 |

**Table S2** LASSO regression analysis for screening variables of cancer-specific survival in metastatic colorectal cancer patients.

| Variables | Multivariable | | |
| --- | --- | --- | --- |
|  | HR | 95%CI | P-value |
| Age |  |  |  |
| ≤60 | - | - |  |
| ＞60 | 1.46 | 1.27,1.67 | <0.001 |
| Primary.Site |  |  |  |
| Proximal colon | - | - |  |
| Distal colon | 0.60 | 0.44,0.81 | <0.001 |
| Rectum | 0.47 | 0.37,0.61 | <0.001 |
| Grade |  |  |  |
| I-II | - | - |  |
| III-IV | 1.60 | 1.33,1.91 | <0.001 |
| Surg |  |  |  |
| No | - | - |  |
| Yes | 0.37 | 0.31,0.44 | <0.001 |
| T |  |  |  |
| T1 | - | - |  |
| T2 | 0.64 | 0.42,0.97 | 0.036 |
| T3 | 0.72 | 0.55,0.95 | 0.019 |
| T4 | 0.97 | 0.73,1.29 | 0.8 |
| N |  |  |  |
| N0 | - | - |  |
| N1 | 1.15 | 0.98,1.36 | 0.095 |
| N2 | 1.59 | 1.31,1.94 | <0.001 |
| bone metastasis |  |  |  |
| No | - | - |  |
| Yes | 1.68 | 1.32,2.13 | <0.001 |
| brain metastasis |  |  |  |
| No | - | - |  |
| Yes | 1.84 | 1.15,2.96 | 0.012 |
| lung metastasis |  |  |  |
| No | - | - |  |
| Yes | 1.37 | 1.17,1.61 | <0.0001 |
| Treat |  |  |  |
| CRT | - | - |  |
| CRT+IMT | 0.54 | 0.44,0.66 | <0.001 |


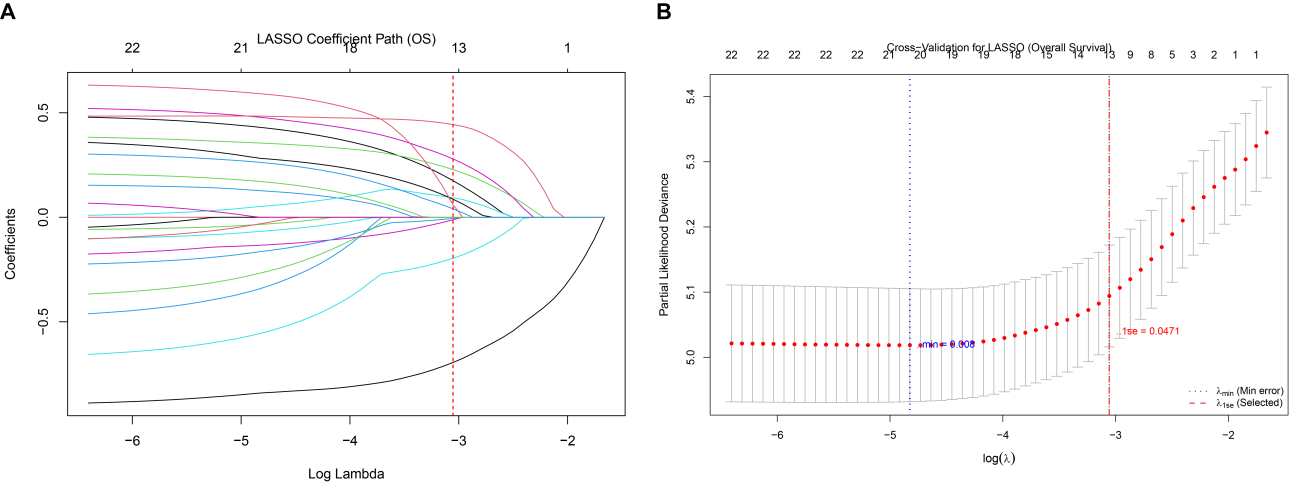


**Figure S1** LASSO coefficient path plot for OS. Each curve represents the shrinkage path of the coefficient of a candidate variable. The vertical dashed red line indicates the optimal penalty (λ.1se) selected by 10-fold cross-validation. Variables with non-zero coefficients at this λ were retained for the final multivariable Cox model of OS(A). Cross-validation curve for LASSO regression of OS. The dashed red line (λ.1se) represents the optimal penalty parameter that yields the most parsimonious model within one standard error of the minimum cross-validation error (λ.min, blue dashed line)(B).


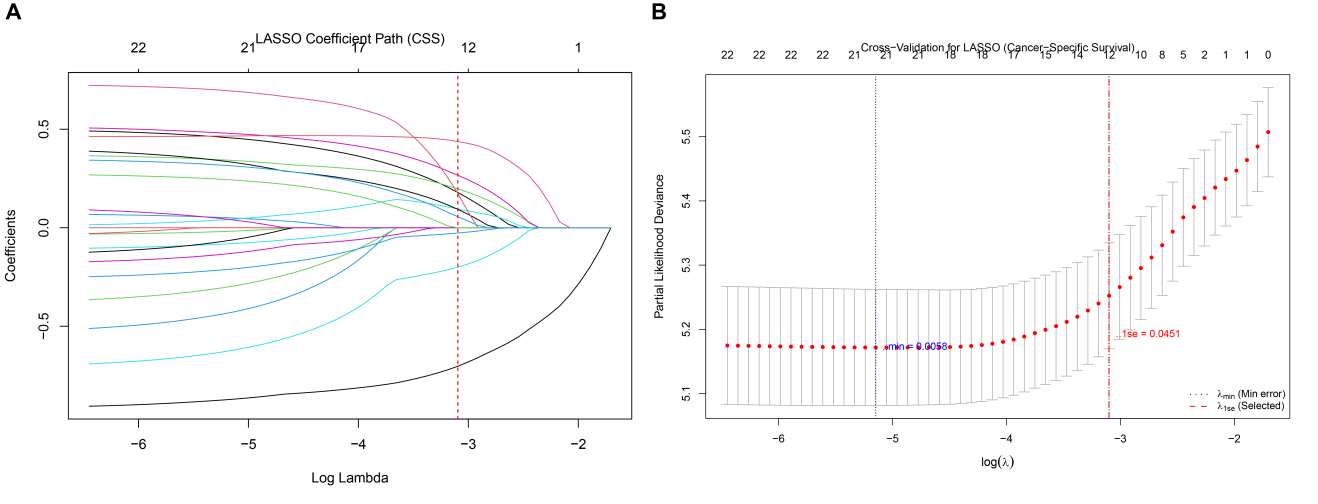


**Figure S2** LASSO coefficient path plot for CSS. The vertical dashed red line indicates the optimal penalty (λ.1se). Variables with non-zero coefficients at this λ were retained for the final multivariable Cox model of CSS(A). Cross-validation curve for LASSO regression of CSS. The optimal penalty parameter (λ.1se, red dashed line) is selected for model parsimony(B).
